# An unexpected cause of sacroiliitis in a patient with gout and chronic psoriasis with inflammatory arthritis: a case report

**DOI:** 10.1186/s12891-018-2044-4

**Published:** 2018-04-20

**Authors:** Safi Alqatari, Roberta Visevic, Nina Marshall, John Ryan, Grainne Murphy

**Affiliations:** 1 Royal College of Physicians of Ireland, Cork University Hospital, Cork, Ireland; 2 Rheumatology Department, Cork University Hospital, Cork, Ireland; 3 Radiology Department, Cork University Hospital, Cork, Ireland

**Keywords:** Axial gout, Inflammatory back pain, Dual Energy Computed Tomography, Sacroiliitis, Spondyloarthritis

## Abstract

**Background:**

Inflammatory back pain is a condition characterized by inflammation of the sacroiliac joints and lower spine. It is frequently seen in patients with spondyloarthropathies like ankylosing spondylitis, psoriatic arthritis, enteropathic arthritis and reactive arthritis. Inflammatory back pain can be caused by many other conditions like infection and crystal deposition such as gout. In this case, it is difficult to specifically identify gout as a cause by ordinary imaging like magnetic resonance imaging (MRI) or ultrasound.

**Case presentation:**

This case report describes a young man with severe psoriasis, presumptive psoriatic spondyloarthropathy and chronic extensive tophaceous gout which was difficult to treat because of non-compliance with medications and lifestyle. He presented with inflammatory type low back and buttocks pain with raised inflammatory markers. MRI of the lower back and sacroiliac joints showed features of active sacroiliitis. He was subsequently treated with a Tumor Necrosis Factor (TNF) alpha inhibitor for presumed axial psoriatic arthritis and had no significant benefit. Two attempts DECT of the lumbar spine was not executed correctly. CT lumbar spine and SIJs showed L2/3 endplate and left SIJ erosions mostly related to gout. Rasburicase was introduced. The tophi decreased in size peripherally with marginal improvement in back pain. From this study, we want to bring to the attention of physicians that gout can lead to back pain with inflammatory changes on MRI. We also want to address the importance of other imaging modalities if the cause of the back pain is not clear.

**Conclusion:**

This case is meant to highlight an important but overlooked cause of active sacroililitis and inflammatory type back pain in patients who have gout, and to bring to the attention that plain X-ray, MRI and ultrasound cannot differentiate between inflammatory sacroiliitis caused by seronegative arthritis versus gouty arthritis. CT scan can add more information but DECT is the preferred method for differentiation and identification of axial tophaceous gout.

## Background

Inflammatory back pain (IBP) is a condition characterized by inflammation of the sacroiliac joints and lower spine. It is frequently seen in patients with spondyloarthropathies like ankylosing spondylitis, psoriatic arthritis, enteropathic arthritis and reactive arthritis. Its primary features include: relatively young age at onset, usually before the age of 40; morning stiffness; back pain present for at least three months or more, and pain relieved by movement (Braun 2010) [1]. IBP can be caused by other conditions like infection and crystal deposition such as gout. In this case, it difficult to specifically identify gout as a cause by ordinary imaging like MRI or US. This case report describes a young man with severe psoriasis, presumptive psoriatic spondyloarthropathy based on symptoms, raised inflammatory markers and MRI results, with treatment failure. He also has chronic extensive tophaceous gout which was difficult to treat because of non-compliance with medications and lifestyle.

### Case Presentation

A 38-year-old man who was previously known to have diffuse skin and nail psoriasis and difficult to manage polyarticular tophaceous gout presented with severe pain in the lower back and buttocks associated with significant stiffness. He had symptoms consistent with inflammatory back pain, associated with a marked diurnal variation, pronounced stiffness and good response to non-steroidal anti-inflammatory drugs (NSAIDs). He had no history of uveitis, inflammatory bowel disease or other extra-articular symptoms. He was non-compliant with urate lowering therapy due to gastrointestinal intolerance.

Examination revealed an obese man with extensive psoriasis covering extensor surfaces of elbows and knees as well as scalp, ears and face. There were tophi in his hands, elbows and feet. He had symmetrical polyarthritis in his hands with a decrease in grip strength. Spinal examination showed a significant decrease in neck movements and lateral spinal flexion bilaterally with a positive modified schober’s test and tender sacroiliac area bilaterally. Chest, heart and abdomen examination are unremarkable. Blood tests done (Table 1).

**Table 1:** Test results

Test	Result
WBCs	9000/L (4,400–11,300/L)
Hemoglobin	14.1 g/dl (11.6–14.5 g/dl)
Platelets	192,000/L (140,000–440,000/L)
Renal Function	Normal
Urate	631 mmol/l
Calcium	2.4 mmol/L (2.1–2.65 mmol/L)
ESR	51 mm (0–10 mm/h)
CRP	23.3 mg/dl (0–5 mg/dl)
HLA-B27	Negative
24-h urine creatinine	1299 mg/day (1000–2000 mg/day)
24-h urine urate	340 mg/day (250–650 mg/day)
24-h urine oxalate	28 mg/day (16–49 mg/day)

**Fig. 1:**
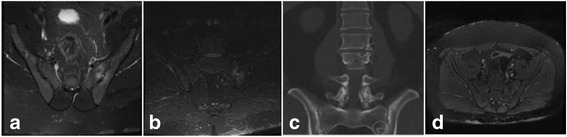
**a** MRI SIJs (axial) pre-treatment T2 FS demonstrating T2 hyperintense synovitis and marrow oedema **b** MRI SIJs (coronal) pre-treatment STIR demonstrating acute left sacroiliitis **c** Conventional CT lumbar spine and SIJs (coronal) with arrows on the L2/3 endplate erosions and circle at the LEFT SIJ erosions **d** MRI pelvis (axial) post-treatment T2 FS demonstrating resolution of T2 hyperintense synovitis and marrow oedema

Lumbosacral spine and sacroiliac joints (SIJs) MRI showed bilateral chronic bilateral sacroiliitis (Fig. 1a) with superimposed acute left sacroiliitis (Fig. 1b). Severe facet joint degenerative changes in the lower lumbar spine with no neural compression was reported in addition.

Our presumed diagnosis was bilateral sacroililitis secondary to psoriatic arthritis, and the patient was started on adalimumab, a tumor necrosis factor alpha blocker (TNF alpha) therapy. Adalimumab was switched to intravenous infliximab 5 mg/kg after 1 month because the patient was struggling with self-administration. Infliximab was titrated up to 7 mg/kg and given for a duration of 4 months. It helped with the patient’s psoriasis but it had no impact on the low back or buttock pain and stiffness. Due to lack of response to TNF alpha treatment and the fact that the patient had several gout flares, the initial diagnosis was questioned. A dual energy computerised tomography (DECT) of the SIJs was arranged but despite two attempts the DECT of the lumbar spine was not executed correctly. However, CT lumbar spine and SIJs showed L2/3 endplate and left SIJ erosions mostly related to gout (Fig. 1c).

Consequently, the patient was given rasburicase on three occasions which decreased the size and number of peripheral joint tophi, but unfortunately had minimal effect on his low back symptoms despite MRI evidence of resolution of T2 hyperintense synovitis and marrow oedema after receiving the treatment (Fig. 1d). His serum urate level and imaging results has responded appropriately and therapy is currently ongoing.

## Discussion

Although spondyloarthropathies are the commonest cause of a sacroiliitis, especially in the younger male population, other conditions like infection as tuberculosis and crystal-induced sacroiliitis can be causal and need to be considered [2, 3]. Although common as a cause of peripheral monoarthritis or oligoarthritis, gout rarely comes to mind when a patient is experiencing lower back pain [2]. It is described in several case reports. Overlap of psoriatic arthritis with gout, causing acute sacroiliitis is possible, but rarely described in literature [4].

In this case, gout coexisting with extensive psoriasis was diagnosed. The patient’s polyarthritis affected multiple small and large joints. In addition to psoriatic plaques, he had large numbers of tophi. There was lower back morning stiffness, of more than two hours duration, which commenced in his twenties shortly after his first presentation with gout. His young age suggests a hereditary predisposition to gout in addition to lifestyle factors like alcohol.

This case highlights the utility of alternative imaging modalities in patients who present or respond atypically to treatment like simple CT scan or DECT. It offers particular benefit in patients with crystal based disease where MRI can be non-discriminative.

DECT is novel but specific investigation**.** DECT if used appropriately with simultaneous acquisition at two energy levels (80 and 140 kVp) allows assessment of difference in tissue absorption and thus a non-invasive determination of chemical tissue composition [5]. Clinical examination is challenging in patients with advanced and mixed disease. It can be difficult to determine if gout exists alone or with other arthropathies. DECT is helpful in diagnosis, monitoring progression and offering an opportunity to determine response to treatment where examination is limited by significant deformity [6]. X-ray, Ultrasound and MRI are non-specific modalities.

Treatment is challenging due to high volumes of tophi and compliance. Pegloticase is perhaps more useful in the longer term [7], this is not available locally at present and therefor ongoing treatment with rasburicase is planned for this gentleman.

## Conclusion

The aim of this case report is to highlight an important but overlooked cause of active sacroililitis and inflammatory type back pain in patients who have tophaceous gout, and to bring to the attention that plain X-ray, MRI and ultrasound cannot differentiate between inflammatory sacroiliitis caused by seronegative arthritis versus gouty arthritis. DECT is the preferred method for differentiation and identification of axial tophaceous gout.

## Abbreviations

CRP: C-reactive protein
DECT: Dual energy computed tomography
ESR: Erythrocyte sedimentation rate
IBP: Inflammatory back pain
MRI: Magnetic resonance imaging
NSAIDs: Non-steroidal anti-inflammatory drugs
TNF: Tumor necrosis factor
US: Ultrasound
WBC: White blood cells
